# Decoding Remapped Spatial Information in the Peri-Saccadic Period

**DOI:** 10.1523/JNEUROSCI.2134-23.2024

**Published:** 2024-06-13

**Authors:** Caoimhe Moran, Philippa A. Johnson, Ayelet N. Landau, Hinze Hogendoorn

**Affiliations:** ^1^ Melbourne School of Psychological Sciences, The University of Melbourne, Parkville, Melbourne, Victoria 3052, Australia; ^2^Department of Psychology,Hebrew University of Jerusalem, Mount Scopus, Jerusalem 9190501, Israel; ^3^ Cognitive Psychology Unit, Institute of Psychology & Leiden Institute for Brain and Cognition, Leiden University, Leiden 2333 AK, The Netherlands; ^4^Department of Cognitive and Brain Sciences, Hebrew University of Jerusalem, Mount Scopus, Jerusalem 9190501, Israel; ^5^ School of Psychology and Counselling, Queensland University of Technology, Kelvin Grove, Queensland 4059, Australia

**Keywords:** EEG, eye movements, multivariate pattern analysis, saccadic remapping

## Abstract

It has been suggested that, prior to a saccade, visual neurons predictively respond to stimuli that will fall in their receptive fields after completion of the saccade. This saccadic remapping process is thought to compensate for the shift of the visual world across the retina caused by eye movements. To map the timing of this predictive process in the brain, we recorded neural activity using electroencephalography during a saccade task. Human participants (male and female) made saccades between two fixation points while covertly attending to oriented gratings briefly presented at various locations on the screen. Data recorded during trials in which participants maintained fixation were used to train classifiers on stimuli in different positions. Subsequently, data collected during saccade trials were used to test for the presence of remapped stimulus information at the post-saccadic retinotopic location in the peri-saccadic period, providing unique insight into when remapped information becomes available. We found that the stimulus could be decoded at the remapped location ∼180 ms post-stimulus onset, but only when the stimulus was presented 100–200 ms before saccade onset. Within this range, we found that the timing of remapping was dictated by stimulus onset rather than saccade onset. We conclude that presenting the stimulus immediately before the saccade allows for optimal integration of the corollary discharge signal with the incoming peripheral visual information, resulting in a remapping of activation to the relevant post-saccadic retinotopic neurons.

## Significance Statement

Each eye movement leads to a shift of the visual world across the retina, such that the visual input before and after the eye movement does not match. Despite this, we perceive the visual world as stable. A predictive mechanism known as saccadic remapping is thought to contribute to this stability. We use a saccade task with time-resolved EEG decoding to obtain a fine-grained analysis of the temporal dynamics of the saccadic remapping process. Probing different stimulus-saccade latencies and an array of stimulus locations, we identify when remapped information becomes available in the visual cortex. We describe a critical window in which feedforward visual information and the preparatory motor signals interact to allow for predictive remapping of a stimulus.

## Introduction

Saccadic eye movements lead to shifts of the visual world across the retina, such that a visual object is represented by different populations of visual neurons before and after the saccade. Despite this, we perceive a stable visual world, undisrupted by intervening saccades. This spatial constancy is thought to be achieved through the combination of predictive information about the impending saccade and sensory information from the visual system (Helmholtz, 1867; Hallett and Lightstone, 1976; Bays and Husain, 2007; Medendorp, 2011). In this interpretation, just before the saccade, visual neurons rapidly shift or “remap” visual stimuli from their current retinotopic position to the future retinotopic position (i.e., their position on the retina after a saccade). It can be thought of as a predictive “glance” at the post-saccadic retinotopic scene before the eyes leave the current fixation.

The generally accepted explanation of predictive remapping is that, when a saccade is initiated, an internal copy of the motor command, termed the corollary discharge, is sent to sensory systems. This contains information about the direction and amplitude of the upcoming saccade, so that the change in sensory input can be predictively anticipated (Sommer and Wurtz, 2002). Predictive remapping has been demonstrated in a range of experimental paradigms. Early research used single unit recordings in monkeys to shed light on the predictive processes underwriting active vision. Researchers found that during saccade planning, which occurs 200 ms prior to saccade onset (Fischer and Ramsperger, 1984), a single neuron would start to fire in response to a stimulus presented outside of its spatial receptive field (RF) if the upcoming saccade would shift that stimulus into its RF (Duhamel et al., 1992). This has been observed in numerous brain areas including visual areas V2, V3, and V3A (Nakamura and Colby, 2002), the lateral intraparietal area (LIP; Duhamel et al., 1992; Gottlieb et al., 1998; Kusunoki et al., 2000), frontal eye fields (Bruce and Goldberg, 1985; Thompson et al., 1996; Umeno and Goldberg, 1997, 2001), and the superior colliculus (Mays and Sparks, 1980; Walker et al., 1995). This predictive neuronal firing has been interpreted as a shift of neuronal receptive fields prior to saccade onset, allowing stabilization of vision despite perturbations caused by the moving eye.

To maintain visual stability, objects that appear before a saccade must be recognized as the same object once the saccade has landed. Remapping provides a prediction of the post-saccadic visual input so that when the eyes arrive at their intended position, the maintained visual information acts as a template against which post-saccadic input can be compared. A plethora of psychophysical studies have demonstrated remapping in humans, showing the integration of visual information across a saccade (Ganmor et al., 2015; Wolf and Schütz, 2015; Paeye et al., 2017). A single stimulus is flashed in the periphery during saccade preparation. On saccade landing, a second stimulus is flashed at the same spatial position but a different retinal position, due to the saccade. Participants often report these stimuli as being part of the same image. This phenomenon, termed trans-saccadic perceptual fusion, can be explained by the pre-saccadic stimulus being remapped to its retinotopic post-saccadic location such that when the eyes arrive and the post-saccadic stimulus is presented, the two are fused (Paeye et al., 2017). Similarly, remapping in humans has also been shown through masking paradigms. In the same way that two discrete flashed stimuli can be integrated across a saccade, a mask presented at the same retinotopic position as the remapped pre-saccadic stimulus will render the pre-saccadic stimulus invisible (De Pisapia et al., 2010). This is evidence that the visual system assumes continuity across saccades and does not reprocess the scene with each new fixation.

Saccadic remapping has also been characterized as an updating of attention around the time of a saccade. Cavanagh et al. (2010) proposed that the phenomenon of receptive field remapping, as described in animal neurophysiological findings, may be explained by the transfer of attentional states between neurons. The ability of the visual system to remap attention prior to a saccade allows for attentional allocation at the relevant locations, despite disruptive saccades (Marino and Mazer, 2016). Using a double-step saccade task, Rolfs et al. (2011) found anticipatory discrimination facilitation at a stimulus' remapped location prior to a saccade. In other studies, an attention capturing cue is presented during the pre-saccadic period, showing that attention predictively shifts to the remapped location of the cue in anticipation of the saccade landing (Jonikaitis et al., 2013; Szinte et al., 2018). This can lead to increased sensitivity to an orientation change at the remapped location (Szinte et al., 2018), masking of a target stimulus when the mask is presented at the remapped location (Hunt and Cavanagh, 2011) or interference with stimulus identification due to “remapped crowding” (Harrison et al., 2013). However, the updating of attention to the remapped location is not an instantaneous event. A study by Golomb et al. (2008) found that attention lingers at the pre-saccadic retinotopic location of the target stimulus for ∼100–200 ms after saccade landing, known as the retinotopic attentional trace. This has been described as a “soft-handoff” whereby visual information from both the pre-saccadic and post-saccadic location is available to higher-level visual areas (Fabius et al., 2020). Although these studies have been instrumental in demonstrating attentional updating in the peri-saccadic period, to what extent spatially specific stimulus information is transferred to the post-saccadic retinotopic location is still up for debate.

In contrast to the direct demonstration of predictive remapping in animal neurophysiological recordings, neurophysiological evidence for predictive remapping in humans is less clear. Predictive remapping involves a precise shift in spatial positions that occurs only briefly, tightly synchronized to saccade execution. Fully characterizing this process requires greater concurrent spatial and temporal precision than has been offered by conventional noninvasive human neuroimaging approaches. For example, functional magnetic resonance imaging (fMRI) studies, employing cross-hemispheric paradigms, have been a popular choice in investigating remapping (Merriam et al., 2003, 2007; Medendorp et al., 2005). In such paradigms, with each saccade, the visual stimulus crosses the vertical meridian, which means that the stimulus must be remapped from the current contralateral hemisphere to the future contralateral hemisphere (Parks and Corballis, 2010). Several studies have found that a stimulus flashed in the pre-saccadic period elicits activation in the ipsilateral hemisphere which was never directly stimulated (Merriam et al., 2003, 2007; Bellebaum and Daum, 2006; Parks and Corballis, 2010). However, the temporal resolution of fMRI is limited, allowing only a very coarse insight into the time course of neural computations. Understanding the temporal dynamics of the saccadic remapping process is essential to understanding the underlying mechanisms; establishing when the brain has access to relevant visual information, whether it be before (pre-saccadic), during (trans-saccadic), or only after (post-saccadic) the saccade, is necessary to understand how the visual system maintains visual stability.

In order to provide this necessary temporal precision, other studies have attempted to investigate saccadic remapping using electroencephalography (EEG) (Bellebaum and Daum, 2006; Parks and Corballis, 2010; Peterburs et al., 2011). However, the limited spatial precision of EEG has constrained conclusions about spatial remapping to a very coarse scale. For example, Parks and Corballis (2010) examined event-related potentials (ERPs) and found that if a saccade shifted a stimulus from one hemifield to the other, prior to that saccade, there was an increase in positivity in the hemisphere ipsilateral to the visual stimulus. This shows, at a coarse level, remapping of visual space in anticipation of a saccade. Other studies have employed a multivariate pattern analysis (MVPA) approach in which participants made a saccade to either a house or a face (Edwards et al., 2018) or an oriented grating (Fabius et al., 2020) located in the periphery. Edwards et al. (2018) reported that when the stimulus remained the same across the saccadic period, they could decode it 123 ms after saccade offset, whereas when the stimulus was changed in the trans-saccadic period, stimulus decoding only became possible later, at 151 ms post-saccade offset. This decoding advantage suggests anticipation of the post-saccadic input before the eyes have moved (Edwards et al., 2018). Similarly, Fabius et al. (2020) found a post-saccadic hand-off whereby pre-saccadic information was available at the post-saccadic position for ∼100 ms after saccade offset. Importantly, neither of these studies sampled multiple stimulus locations, limiting the conclusions that can be drawn about the spatial specificity of the remapped response. Moreover, both studies involved stimuli that were visible before, during, and after the saccade [Edwards et al. (2018) included one condition in which the stimulus was removed from the screen before the eyes reached the saccade target; this is considered in the discussion section]. Although the interaction between pre-saccadic and post-saccadic input is suggestive of remapping, a direct demonstration of predictive remapping would require evidence that a pre-saccadic visual stimulus is represented neurally at its post-saccadic retinotopic location, uncorrupted by any input once the eyes start moving. This would ensure that any sensory information about the stimulus was acquired before saccade onset and therefore that any neural representation of the stimulus in the remapped location is attributable to predictive remapping.

In this study, we applied an EEG decoding approach to investigate the neural representation of stimulus position in the presence of a saccade. Stimuli were briefly presented before saccade onset and removed before saccade landing. This ensured that there was no direct visual stimulation at the remapped location. This novel analysis approach, in combination with a careful experimental design, allowed us to characterize the fine-grained time course of neural position information for stimuli presented in multiple different positions and at different stimulus-saccade latencies. Using this approach, we show that visual stimuli presented during a narrow temporal window 100–200 ms before a saccade are subsequently represented in the visual cortex in their future post-saccadic retinotopic positions, providing strong neural evidence of predictive remapping in humans.

## Materials and Methods

### Participants

Participants were required to complete six testing sessions across different days, including one screening session. The screening session provided a sanity check to ensure above-chance decoding of stimulus position on fixation trials. This was used as an inclusion criterion. In Protocol 1, 15 observers took part in the initial screening. Six participants were excluded after analysis of their screening session data, due to poor EEG classification performance (<52% average decoding accuracy when classifying stimulus presentation location on fixation trials), and an additional participant was excluded after completing all six sessions due to data collection errors. After exclusion, eight observers (four male; mean age, 26.1 years; SD = 5.5 years) with normal or corrected-to-normal vision remained. In Protocol 2, 13 observers took part in the initial screening session, of whom three were excluded due to poor EEG classification performance (<52% average decoding accuracy when classifying stimulus presentation location on fixation trials). After exclusion, 10 observers (three male; mean age, 25.7 years; SD = 2.0 years) with normal or corrected-to-normal vision remained. Participant data was combined across the protocols giving a total of 18 participants (seven male; mean age, 26.4 years; SD = 4.1 years). The experimental protocols were approved by the human research ethics committee of The University of Melbourne, Australia (Ethics ID: 2021-12985-16726-4) and conducted in accordance with the Declaration of Helsinki. All observers provided informed consent before beginning the experiment and were reimbursed AU$15 per hour for their time, plus an additional AU$20 after completing all sessions.

### Stimuli and procedure

Stimuli were programmed in MATLAB version R2020a, using the Psychophysics Toolbox extension (Brainard, 1997; Pelli, 1997; Kleiner et al., 2007). They were presented on an ASUS ROG PG258 monitor (ASUS) with a resolution of 1,920 × 1,080 running at a refresh rate of 120 Hz. Participants were seated in a quiet, dark room with their head supported by a chin rest, positioned 80 cm from the screen.

Stimuli differed slightly across the two protocols of the experiment. In both protocols, stimuli consisted of sinusoidal gratings presented within a circular Gaussian window [i.e., Gabor; outer diameter: 7.9° of visual angle (dva); 100% contrast] presented on a gray background for 100 ms. In Protocol 1, the stimulus was always presented at a spatial frequency of 0.16 c/dva (except on catch trials), while in Protocol 2 the stimulus could appear at a spatial frequency of 0.33 c/dva or 1 c/dva. In Protocol 1, grating orientations were evenly spaced between 0 and 150° in 30° steps, meaning six possible orientations could be presented (stimuli were collapsed across orientations for analyses). In Protocol 2, orientation of the grating was fixed at 0° (except on catch trials). For catch trials, participants were instructed to report an oddball grating of either a higher spatial frequency; 0.51 c/dva, (Protocol 1) or of a different orientation; 90° (Protocol 2) which could appear every 11–20 trials. These trials were included in order to ensure attentive processing of the grating stimuli and were excluded from the main analysis. All other details were the same across the two experimental protocols.

Two fixation points (0.41 dva), one black and one white, were horizontally aligned and subtended 10.04 dva to the left and right of the screen center (20.08 dva apart). They appeared at the beginning of the experiment and remained visible throughout, excluding breaks. Participants were instructed to fixate on the black fixation point at all times and to press the space bar when they detected an odd stimulus. Periodically during the experiment, a saccade cue appeared: the color of the fixation points gradually changed, such that over a period of 1.2 s the black fixation point became white and vice versa. Participants were instructed to monitor the color of the fixated fixation point and plan and execute a saccade from one fixation point to the other as soon as they detected the color change, such that they were always fixating the black fixation point.

**Figure 1.**
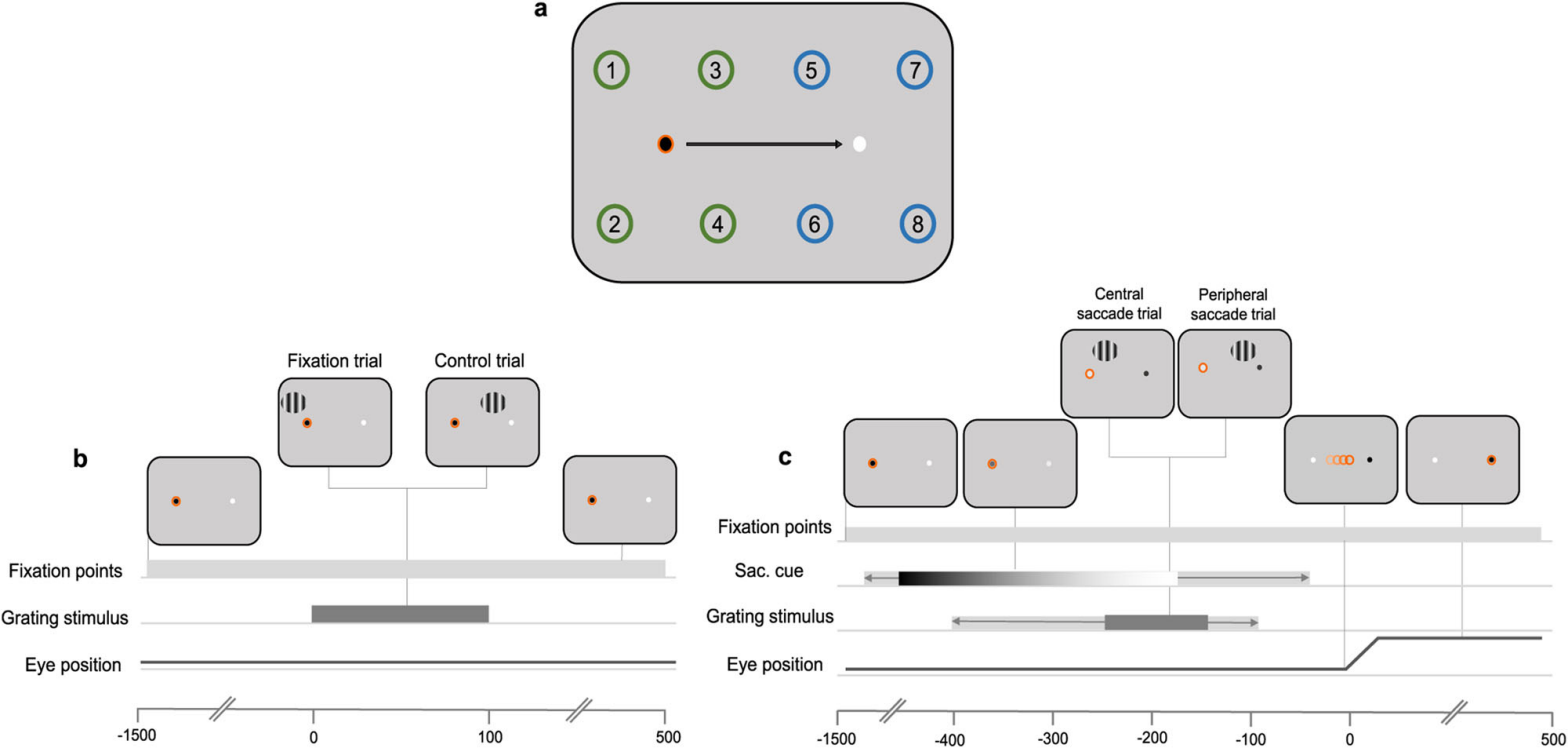
***a***, Stimulus configuration. Participants always fixated on the black fixation point and made a saccade when cued by a gradual color change to white. Stimuli were presented around fixation on fixation trials (locations 1–4) and around the white fixation point on control trials or the saccade target on saccade trials (locations 5–8). The orange circle around the black fixation point indicates eye position. The numbers on the screen indicate all possible stimulus presentation locations. The green and blue circles indicate where stimuli could be presented on fixation trials and control or saccade trials, respectively**.** The black arrow shows saccade direction. All are for illustration purposes only and were not presented to participants. ***b***, In fixation trials, participants maintained fixation on the black fixation point throughout the trial, never fixating the white. The orange ring indicates the position of the eyes. Approximately 400–800 ms after trial onset, the grating stimulus was presented at one of four locations around fixation. The stimulus was displayed for 100 ms. In control trials, participants fixated on the black fixation point. A grating was presented at one of four locations around the alternate fixation point (white circle). ***c***, In saccade trials, participants fixated on the black fixation point. After 200–600 ms, the black fixation point changed to white and the white fixation point changed to black, which served as the saccade cue. The colors gradually changed over a period of 1.2 s, indicated here by the color gradient. The light gray bar behind the gradient refers to possible start/end times of the color change. In a period ranging from 400 to 100 ms before saccade onset, a grating was presented at one of four locations around fixation (central saccade trials) or around the saccade target (peripheral saccade trials). The stimulus was presented for 100 ms. The light gray bar behind the dark gray bar refers to possible start/end times of the stimulus.

Different conditions are determined by the conjunction of fixation position and the location of the grating. In fixation trials, the fixation is stable (no color change occurs), and stimuli appear in one of four positions around the current black fixation point (Fig. 1*a*, locations 1–4). In control trials, the fixation is stable, and stimuli appear on the other side of the screen, in one of four locations around the white fixation point (Fig. 1*a*, locations 5–8). In saccade trials, there is a color change, and stimuli appear during the planning of a saccade. In this case, stimuli can appear around current fixation (central saccade trials; Fig. 1*a*, locations 1–4) or the saccade target (peripheral saccade trials; Fig. 1*a*, locations 5–8; Fig. 1*b*,*c* for task design).

Stimulus locations were separated by 5.06 dva and were also 5.06 dva away from the nearest fixation point (Fig. 1*a*). The grating was presented for 100 ms at a variable delay after the saccade cue. This delay was adjusted for each participant in order to sample trials with ∼200 ms between stimulus onset and saccade onset. To do so, the latency between grating onset and saccade onset was recorded on every trial and averaged over the previous 100 trials (see Fig. 4*b* for the distribution of stimulus-saccade onset latencies).

Each experimental session contained a total of 2,400 trials: 1,120 fixation trials, 1,120 saccade trials, and 160 control trials, randomly interleaved across five blocks. There were a total of 480 trials per block, with a minibreak every 100 trials. Trials were split evenly between fixation and saccade trials (224 trials each) with the remaining allocated to control trials (32 trials).

### EEG and eye-tracking pre-processing

EEG and EOG data were recorded at 2,048 Hz using a BioSemi system, with 64 active electrodes and 6 ocular electrodes. The continuous EEG and eye-tracking data was pre-processed off-line using MATLAB version R2020a and EEGLAB toolbox (v2021.0; Delorme and Makeig, 2004). The data was first down-sampled to 256 Hz and then re-referenced to the mastoids. Eye-tracking data were recorded using an EyeLink 1000 eye-tracker (SR Research) at 1,000 Hz. The eye-tracker was calibrated at the start of the experiment and at the beginning of every block. In addition, drift correction was applied at each minibreak within a block. The eye-tracking data was synchronized with the EEG using the EYE-EEG toolbox version 0.81 (Dimigen et al., 2011). Saccades were detected using a velocity-based algorithm (Engbert and Kliegl, 2003) within the EYE-EEG toolbox. Successive eye positions were considered saccades if the velocity of the left eye exceeded a threshold of 5 standard deviations (median-based) of all recorded eye velocities (excluding blink intervals) for at least 15 ms. A low threshold was used to ensure the detection of microsaccades which are important for the removal of eye movement-related artifacts in later steps (Dimigen et al., 2009). If the time between two saccadic events was <50 ms, only the first saccade was kept in order to avoid including post-saccadic oscillations as separate saccades (Dimigen, 2020). Following this, saccades were considered as valid if they exceeded an amplitude of 15 dva.

The EEG data was then notch filtered at ∼50 Hz to remove electrical artifacts and bandpass filtered between 0.1 and 80 Hz. Automatic data rejection was employed to remove any major artifacts using the Artifact Subspace Reconstruction (ASR) method in EEGLAB. The ASR rejection threshold parameter *k* was set to 15. Bad channels, noted during data collection and confirmed later offline, were spherically interpolated.

To correct for eye movement artifacts in the EEG, we applied independent component analysis (ICA; Makeig et al., 1996). To identify eye movement-related components, the variance ratio of the component activation during periods of eye movements (blinks and saccades) was compared with that during fixation periods (Plöchl et al., 2012). ICA was performed in a separate pre-processing pipeline containing an additional high-pass filter (Hamming windowed sinc FIR, edge of the passband: 2 Hz). It was run on clean continuous data with major movement artifacts removed. The ICA weights were then appended to the corresponding datasets in the original pre-processing timeline and IC activations were recomputed. Components were rejected if the mean variance of activity selected around a saccade (−0.02–0.01 ms) was 10% greater than the mean variance during fixation periods (Plöchl et al., 2012; Dimigen, 2020).

For all trial types, if the gaze deviated >2.5 dva from the current fixation point while the stimulus was on the screen, the trial was removed. Similarly, if the saccade landing was >2.5 dva away from the saccade target, the trial was discarded. In addition, only trials in which the physical stimulus was removed from the screen by the time the eyes arrived at the saccade target were included. Previous studies of trans-saccadic fusion (Wolf et al., 1980; Jonides et al., 1982) have been challenged because the results could be explained by lingering visual monitor persistence (Irwin et al., 1983, 1990). This is not the case in the present study as the gray-to-gray time of the monitor used was 1 ms. Additionally, it has been reported that LCD monitors are optimal when visual persistence is a concern given the short rise times (1–6 ms; Lagroix et al., 2012).

EEG data were epoched for fixation, control, and saccade trials separately. Across all trial types, epochs were time-locked to the presentation of the grating. Epochs were extracted from 200 ms before stimulus onset to 500 ms after and were baseline corrected to the mean of the 100 ms period before stimulus onset. Fixation trials were subsequently included in the training set for the main analysis.

### Multivariate pattern analysis

Epochs from the training set of fixation trials were used to train time-resolved pairwise linear discriminant analysis (LDA) classifiers (Grootswagers et al., 2017) to dissociate the neural activation patterns associated with the presentation of the stimulus at two different positions, using the amplitude from the 64-electrode channels as features. This set of LDA classifiers was tested on each pairwise combination of the four possible stimulus presentation locations on independent fixation trials and subsequently averaged across all pairs. This was done separately for left and right fixation trials, across all timepoints in the epoch and for each individual participant. An above-chance classification performance indicates that the EEG signal contained information that allowed the classifier to distinguish a stimulus presented at one location versus another. The same trained classifiers were used to decode stimulus location on control trials and saccade trials. Pairwise analyses were chosen over a four-way classification to avoid biases caused by multiclass comparisons (Yan et al., 2020).

To examine the time course of remapped stimulus location information, the classifiers that were trained to dissociate stimulus locations on fixation trials were tested using data from the peri-saccadic period of peripheral saccade trials, time-locked to stimulus onset. In a similar manner, central saccade trials were used as a test set to examine the evolution of the pre-saccadic stimulus representation across the peri-saccadic period. As an initial step, trial-by-trial classification time courses were split into three bins depending on when the saccade occurred relative to stimulus onset (i.e., stimulus-saccade onset asynchrony; SSOA), for the purposes of visualization and statistical inference. We sorted saccades into a Short Bin (SSOA = 100–200 ms), a Medium Bin (200–300 ms), and a Long Bin (300–400 ms). “Short,” “medium,” and “long” refer to the time between stimulus onset and saccade onset. In order to probe the precise timing of remapping, we then used a more fine-grained analysis in which a sliding window, with a window length of 100 ms and a step size of 10 ms, was moved along the saccade trial time course.

For classification of peripheral saccade trials, we trained classifiers on the timepoint of peak decoding from fixation trials and tested them on every timepoint of peripheral saccade trials. This approach was taken as the peak timepoint from fixation trial decoding is when stimulus location is most dissociable, i.e., the classifier contains the maximum amount of information about stimulus position. To classify central saccade trials, classifiers were trained on every timepoint of fixation trials, starting from stimulus onset, and tested on corresponding timepoints of saccade trials. This approach was taken as the training and testing set include the same retinotopic stimulus positions. The goal of central saccade decoding is to examine how an imminent saccade affects the representation of the stimulus at its presented location. Therefore, this approach allows us to examine at what timepoint the EEG activation pattern starts to become dissimilar to that of fixation trials.

### Statistical inference

We used Bayes factors (BFs) to determine above- and below-chance decoding and at chance decoding (i.e., null hypothesis) at every timepoint within each of the 18 participants using the Bayes Factor R package (Morey and Rouder, 2018) implemented in Python (Teichmann et al., 2021). We set the prior for the null hypothesis at 0.5 (chance decoding) for assessing the decoding results and at 0 for assessing differences across decoding results. A half-Cauchy prior was used for the alternative hypothesis with a medium width of *r* = √22 = 0.707. Based on Teichmann et al. (2021), we set the standardized effect sizes expected to occur under the alternative hypothesis in a range between −∞ and ∞ to capture above- and below-chance decoding with a medium effect size (Morey and Rouder, 2018).

BFs larger than 1 indicate that there is more evidence for the alternative hypothesis than the null hypothesis (Dienes, 2011) with a BF >3 considered as “substantial” evidence for the alternative hypothesis, i.e., decoding above- or below-chance level. A BF <1/3 is considered substantial evidence for the null hypothesis, i.e., chance-level decoding (Jeffreys, 1939, 1961).

### Task performance

All participants performed well at the oddball task with a mean accuracy of 98% (SE = 2%) in Protocol 1 and 84% (SE = 7%) in Protocol 2. Reaction times (RTs) were calculated from the time that the oddball stimulus appeared on the screen until the time that the space bar was pressed. The mean RT was 670 ms (SE = 128 ms) in Protocol 1 and 623 ms (SE = 150 ms) in Protocol 2.

## Results

### Decoding the position of central stimuli

**Figure 2.**
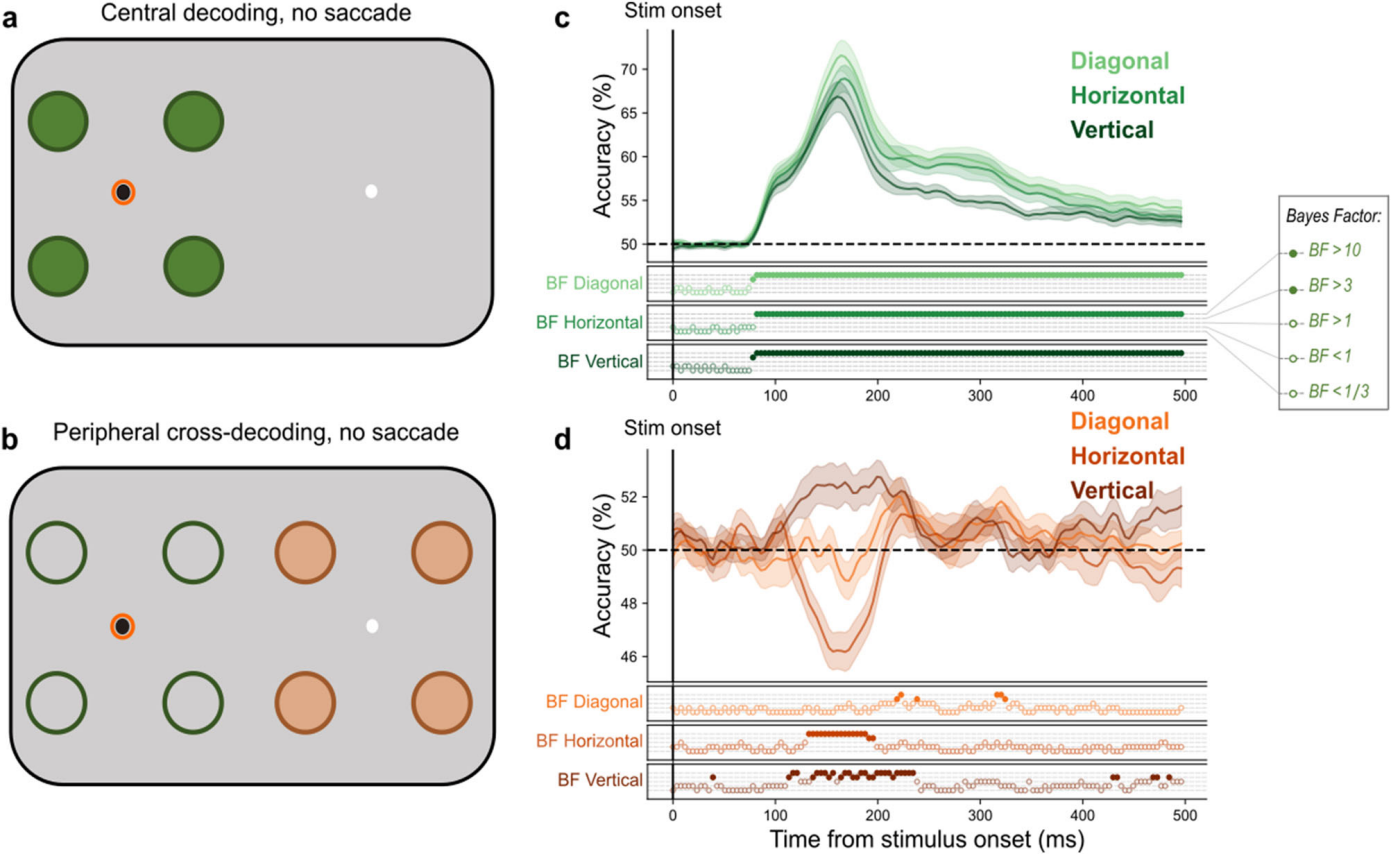
Decoding results across fixation and control trials. This figure illustrates how the classifiers were trained and tested to distinguish neural patterns evoked by the presentation of a stimulus at different locations in (***a***) fixation trials and (***b***) control trials. In ***a*** and ***b***, filled circles represent possible stimulus presentation locations and thus, what the classifiers were tested on. In ***a***, these also correspond with the training locations. The unfilled circles in ***b*** indicate the locations that the classifier was trained on (using fixation data). The bright orange ring indicates current fixation. ***c***, The mean classification over time for fixation trials. The plotted results reflect the average performance at corresponding training and testing timepoints. The timepoint of peak performance for each subject was subsequently used as training data to test control and saccade trials. ***d***, The mean classification over time for control trials. The plotted results reflect the average performance of classifiers trained on the peak timepoint of fixation trials and tested on the entire control trial time course. Shaded areas depict standard error of the mean across subjects. All decoding results are averaged over the relevant pairwise comparisons, across left and right fixation, and subsequently, across subjects. The BFs below the plots indicate the timepoints at which there was substantial evidence in favor of the alternative hypothesis, i.e., decoding above- or below-chance (filled circles). Timepoints at which there was not enough evidence or substantial evidence for the null hypothesis, i.e., chance level, are indicated by open circles.

In order to assess whether we could extract location information from the EEG signal, we trained classifiers on a subset of EEG epochs in which stimuli were presented in the four locations around current fixation and tested classifier accuracy using left out fixation trials (Fig. 2*a*). Using a fivefold cross-validation procedure and pairwise comparisons between stimulus locations, we found that classifiers effectively labeled the test set trials in their correct location across all pairs (Fig. 2*c*). The pairs consisted of diagonal comparisons (i.e., 1 vs 4 and 2 vs 3 in Fig. 1*a*), vertical comparisons (i.e., 1 vs 2 and 3 vs 4; Fig. 1*a*), and horizontal comparisons (i.e., 1 vs 3 and 2 vs 4; Fig. 1*a*). Overall, performance was very similar across the different comparisons, with vertical, diagonal, and horizontal comparisons peaking at 72, 67, and 69% accuracy, at latencies of 164, 160, and 164 ms, respectively (Fig. 2*c*). Together, these results demonstrate that multivariate classifiers were able to extract position information from the EEG signal.

**Figure 3.**
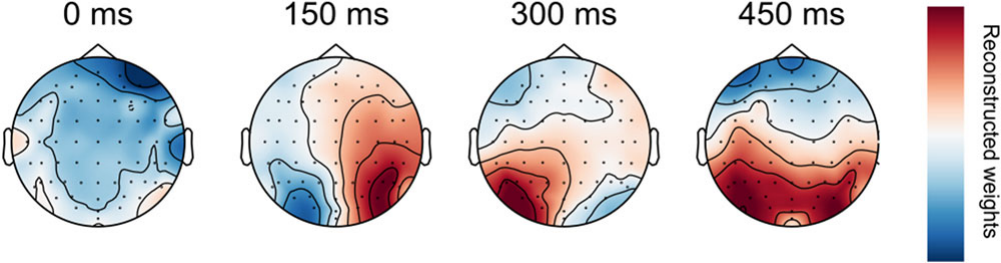
Transformed classifier weights for horizontal comparisons in fixation trials. The timepoints refer to time after stimulus onset. Darker colors indicate channels that contribute positively (red) and negatively (blue) to stimulus location decoding in fixation trials (Haufe et al., 2014).

The decoding topographies for trained classifiers indicate that occipital electrodes contribute most to the above-chance decoding of stimulus position in fixation trials (Fig. 3). This is expected given the visual processing hierarchy. These classifiers were used to test control and saccade trials in subsequent analyses. Thus, successful dissociation of stimulus position by the classifier in control and saccade trials is likely driven by the occipital electrodes

### Generalizing position decoding to peripheral stimuli

After establishing that classifiers were able to decode the position of a stimulus on fixation trials, we investigated whether position information could be decoded from peripheral stimuli in the absence of a saccade (Fig. 2*b*). To do so, we tested these same classifiers on control trials, in which the stimulus was presented in the periphery but no saccade was made. Accordingly, the same classifiers that were trained to discriminate pairs of stimulus locations on fixation trials were tested using EEG data collected during control trials, with locations counted as “correct” if the retinotopic training position matched the retinotopic position of the control stimulus, relative to the other fixation point (i.e., 1–5, 2–6, 3–7, 4–8 in Fig. 1*a*). The peak timepoint from the trained classifiers (fixation trial data) was tested on every timepoint of control trials (0–500 ms after stimulus onset). Decoding was done on an individual subject level, meaning the training timepoint used varied across subjects. The mean peak training timepoint was 167 ms (SE = 24 ms) after stimulus onset.

We observed above-chance cross-decoding for both vertical and diagonal pairwise comparisons and below-chance cross-decoding for horizontal pairwise comparisons (Fig. 2*d*). Above-chance decoding in the vertical comparisons indicates that even though classifiers were trained on stimuli near fixation and tested on stimuli in the periphery, central and peripheral stimuli shared EEG features. This is understandable since ERPs evoked by stimuli in the upper and lower visual fields are highly dissociable (Srebro, 1985; Slotnick 2018). Similarly, diagonal comparisons were above chance for control trials (albeit briefly), due to the upper/lower visual field dissociation. Conversely, we observed below-chance decoding performance for horizontal comparisons. This is most likely attributable to the relative spatial proximity of the mismatched spatial positions. For example, a classifier trained to discriminate between positions 1 and 3 (Fig. 1*a*) and tested on control position 5 is more likely to assign that trial to position 3 than to position 1 due to its relative proximity. This assignment is opposite to the retinotopic locations of the stimuli relative to the two fixation points (i.e., 3 is upper-right in the training set, relative to current fixation, while 5 is upper-left in the test set, relative to the alternate fixation point).

Importantly, because we observed above-chance decoding for control stimuli separated by the horizontal midline (i.e., vertical and diagonal comparisons), only horizontal comparisons were used in subsequent steps to investigate saccadic remapping. This is appropriate because it ensures the most conservative possible approach to demonstrating remapping: without a saccade, decoding performance is significantly below chance at the putative remapped location.

### Generalizing position decoding to peripheral stimuli with a saccade

**Figure 4.**
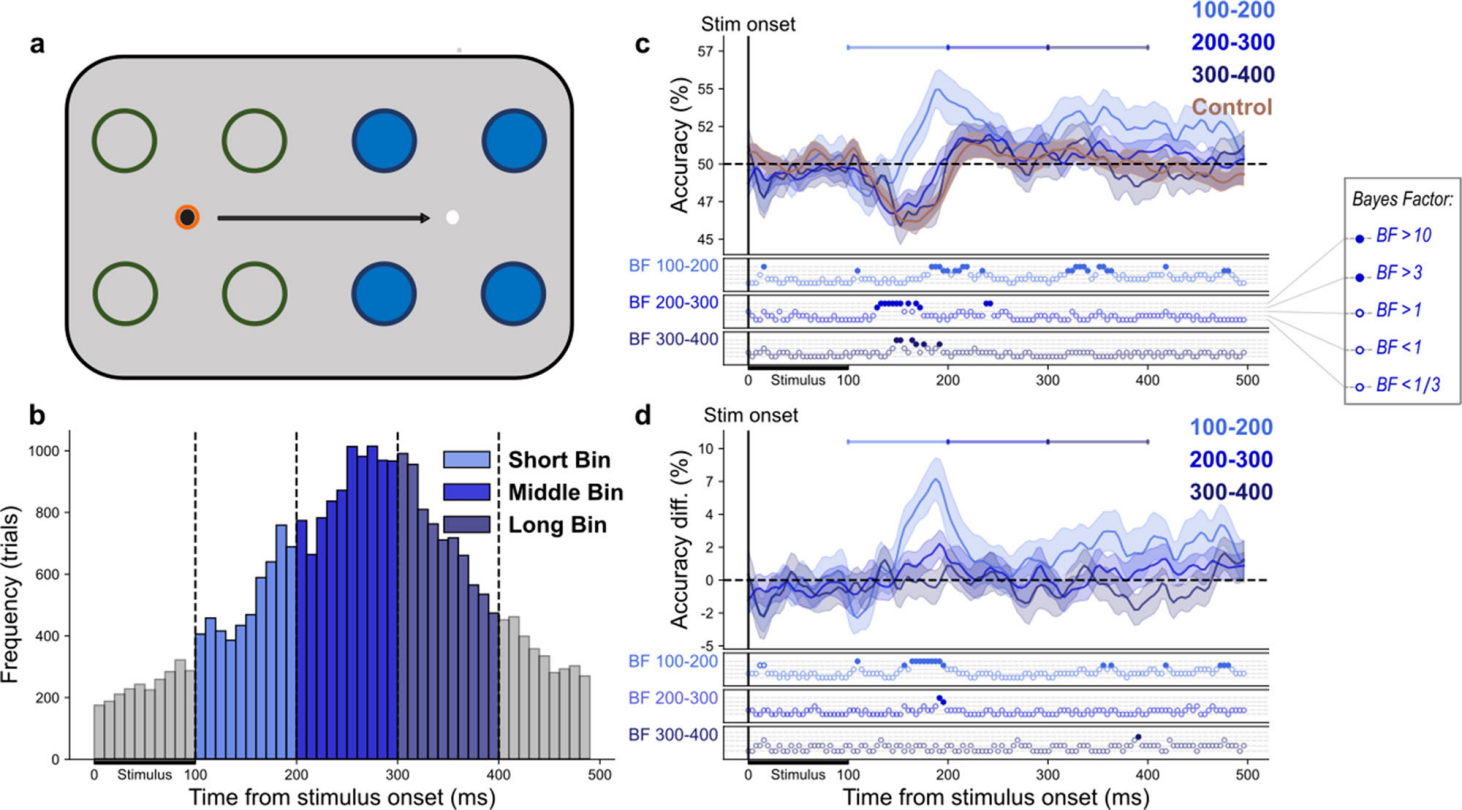
Peripheral saccade trial analyses. ***a***, Classifier training and testing for “peripheral” saccade trials. The unfilled green circles indicate the locations that the classifiers were trained on (using fixation data). Filled blue circles represent possible stimulus presentation locations and thus, what the classifiers were tested on. The black arrow indicates the planned saccade. The bright orange ring indicates current fixation. ***b***, Distribution of saccade latencies relative to stimulus onset are shown and split into three 100 ms bins (Short Bin: 100–200 ms, Middle Bin: 200–300, and Long Bin: 300–400). The horizontal black bar from 0 to 100 ms indicates the time that the stimulus was on the screen. The gray bins were not considered in this analysis. ***c***, Average performance of classifiers trained on the peak timepoint of fixation trials and tested on control and “peripheral” saccade trials. In this case, classification assignment is considered correct if the stimulus is assigned to the corresponding remapped position around the black fixation point. Different shades of blue represent different saccade latency bins, and brown indicates control trials. The BFs below the plots indicate the timepoints at which there was substantial evidence in favor of the alternative hypothesis, i.e., decoding above- or below-chance (filled circles). Timepoints at which there was not enough evidence or substantial evidence for the null hypothesis, i.e., chance level, are indicated by open circles. ***d***, The difference in classification accuracy between the control condition and all saccade latency bins. Only in the Short Bin were there consistently any timepoints in which there was sufficient evidence of a difference between the saccade condition and the control condition. The BFs below the plots indicate the timepoints at which there was substantial evidence of a difference in classification accuracy between saccade and control trials (filled circles) or no difference (unfilled circles). In ***c*** and ***d***, shaded areas depict standard error of the mean across subjects. All decoding results are averaged over the relevant pairwise comparisons, across left and right fixation, and, subsequently, across subjects.

To investigate whether spatial information is remapped in the peri-saccadic period, we next tested the same fixation classifiers on peripheral stimuli presented during saccade trials (Fig. 4*a*). To gain a better understanding of the unfolding of events from stimulus presentation until after the saccade, we split the saccade trials into three different bins based on SSOA. The mean saccade latency for the Short Bin (100–200 ms) was 157 ms (SD = 29 ms), for the Middle Bin (200–300 ms) was 254 ms (SD = 28 ms), and for the Long Bin (300–400 ms) was 344 ms (SD = 29 ms; Fig. 4*b*). The same trained classifier (horizontal comparisons in fixation trial data) was tested on the entire saccade trial (0–500 ms after stimulus onset) across bins. As in the control analysis, classifiers were trained on the timepoint of peak decoding in fixation trials for each participant.

Medium and long latency bins followed a similar pattern of decoding to that seen in control trials, with a below-chance classification peak at 145 ms (47%) and 152 ms (45%), respectively (Fig. 4*d*). As in control trials, this is likely due to the “misclassification” of the stimulus as being at the position closest to the veridical stimulus location and not at the remapped location (which was considered the “correct” location). For example, a stimulus presented at position 5 would be misclassified as being at position 3 (closest spatiotopic position) rather than position 1 (remapped position; Fig. 1*a*). This pattern of results suggests the absence of remapping. The classifier labels the peripheral stimulus as being at the nearest trained spatiotopic position as it likely elicits the most similar EEG pattern. This indicates that the peripheral stimulus is represented at its true retinotopic location in the periphery and not at the remapped location.

In contrast, the Short Bin showed the opposite effect, first rising above chance level at 184 ms with peak decoding (55%) at 188 ms post stimulus onset (Fig. 4*c*). Above-chance decoding means that the pattern of EEG activation for peripheral saccade trials, in which the stimulus occurred in close proximity to saccade onset, was similar to that elicited by a stimulus presented during fixation trials, at a location corresponding to the remapped location. For example, a stimulus presented at location 5 in the Short Bin elicits a similar pattern of EEG activation to a stimulus presented at location 1 on fixation trials (Fig. 1*a*). We then looked at the difference between decoding accuracy for each of the three saccade bins relative to control trials (Fig. 4*d*). Only the shortest latency bin was consistently and significantly different from control trials, with a peak difference of 9% at 188 ms post-stimulus onset.

### Fine-grained temporal analysis

**Figure 5.**
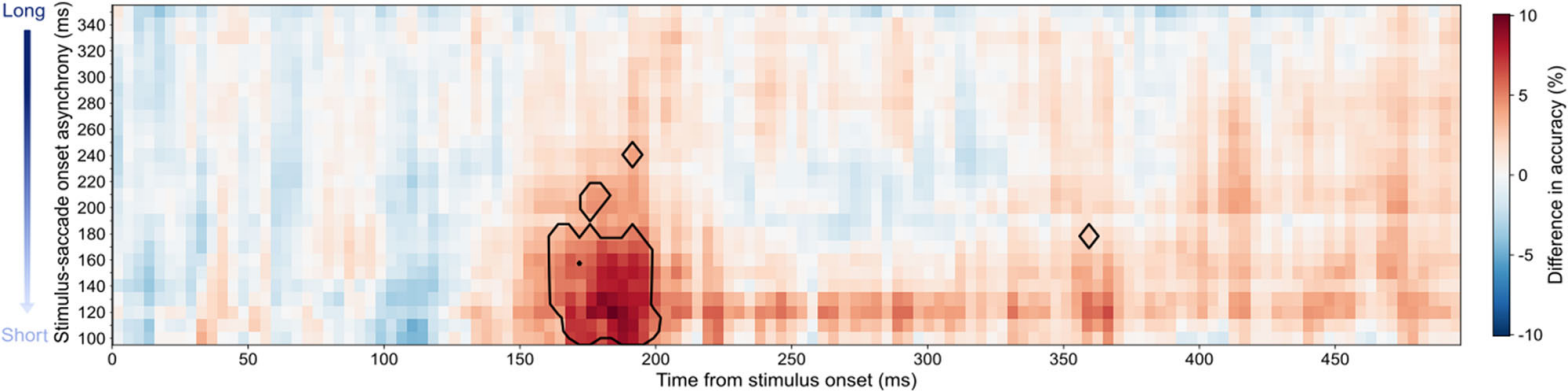
Difference in classification accuracy between control trials and peripheral saccade trials. The classification results of control trials were subtracted from each saccade bin to provide accuracy differences. The *y*-axis shows saccade bin centers ranging from long to short SSOAs, i.e., the time between stimulus onset and saccade onset. The classification results for each of the 26 bins occupy a single row in the matrix. The areas outlined in black indicate a BF >10.

Analyses so far have shown that spatial information is remapped for trials in which a stimulus is presented roughly 100–200 ms before a saccade. To further investigate precisely when a saccade must be made for remapping to be evident, and when the remapped information becomes available in the EEG signal, we carried out a further analysis. We again trained classifiers on the peak timepoint of fixation trials (by subject). While saccade trials were previously split into three nonoverlapping bins, this time we split the trials into overlapping bins, defined using a sliding window that moved from 400 to 50 ms before saccade onset. Thus, the bins evolve from long SSOAs to short SSOAs. Each bin was 100 ms long and the window was shifted by 10 ms each time, giving a total of 26 bins (Fig. 5). Evidence in favor of remapped spatial information (BF > 10) emerged in the early cluster of bins ranging from a bin center of 100–180 ms. The largest difference in decoding accuracy (10%) between control and saccade trials occurred in the bin ranging from 70 to 170 ms (bin center, 120 ms) at 184 ms post-stimulus onset. This indicates that spatial information was remapped only when a saccade occurred ∼100–180 ms after stimulus onset, with strongest evidence for remapping when a saccade occurred ∼120 ms after stimulus onset.

### Position decoding of central stimuli when a saccade is imminent

**Figure 6.**
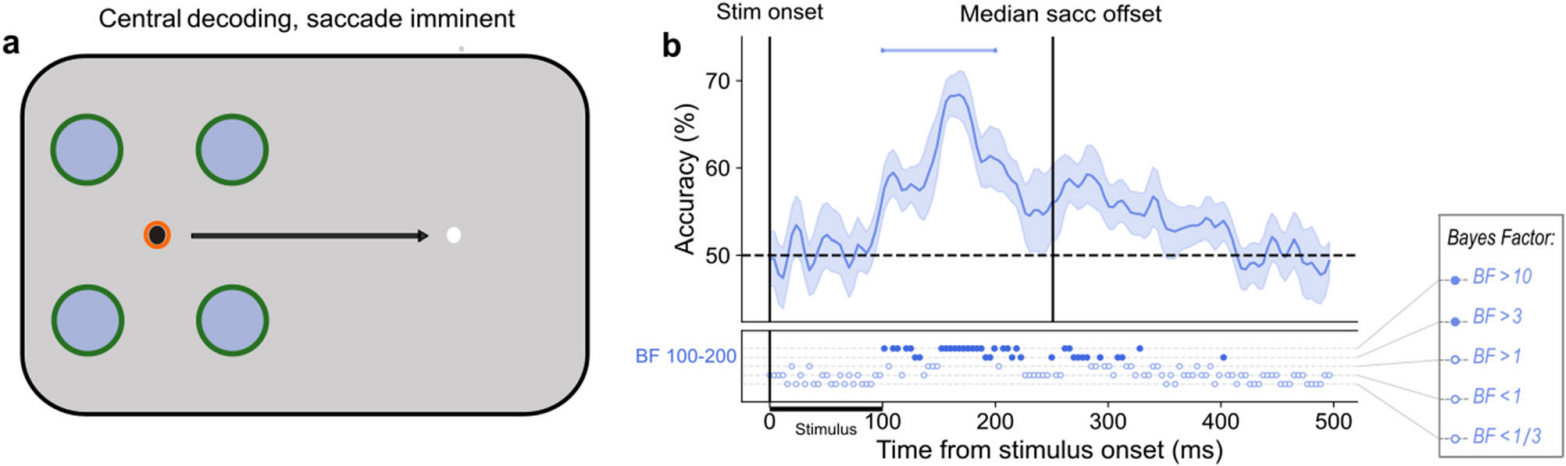
Central saccade trial analysis. ***a***, Classifier training and testing for “central” saccade trials. The green rings signify the locations that the classifier was trained on (using fixation data). The blue filling indicates the possible stimulus presentation locations and thus, what the classifiers were tested on. Therefore, in this analysis the training locations and testing locations overlapped. ***b***, Average performance of classifiers trained on fixation trials and tested on corresponding timepoints of “central” saccade trials. The horizontal blue line indicates the range of saccade onsets. The median saccade offset occurs at 254 ms post-stimulus onset. The BFs below the plot indicate the timepoints at which there was substantial evidence in favor of the alternative hypothesis, i.e., decoding above-chance (filled circles). Timepoints at which there was not enough evidence or substantial evidence for the null hypothesis, i.e., chance level, are indicated by open circles.

To understand how the representation of a stimulus' pre-saccadic retinotopic location is altered as a result of remapping, we tested the fixation classifiers using central saccade trials. These are trials in which the stimulus was presented at one of the four locations around current fixation while the participant prepared a saccade (Fig. 6*a*). Only saccade trials that fell within the Short Bin (100–200 ms) were included as remapping was found at this SSOA. The mean saccade latency was 161 ms (SD = 26 ms). Classifiers were trained on the entire fixation trial period and tested on corresponding timepoints in “central” saccade trials.

This analysis revealed two main findings. The first is that the classifier could decode the stimulus location well above chance (peak, 70%) at timepoints (183–188 ms) overlapping those at which the remapped location was decodable in peripheral saccade trials. This suggests that both the remapped stimulus location and the pre-saccadic stimulus location are simultaneously represented in the trans-saccadic period. The second result is that the classifier could still decode the stimulus' retinotopic pre-saccadic location up to 100 ms after saccade offset [cluster BF > 3; from median saccade offset (254 ms) to 384 ms post stimulus onset; Fig. 6*b*]. This indicates that the pre-saccadic location is maintained across the saccade and is available into the new fixation.

## Discussion

Despite the constant movement of our eyes, we are able to keep track of relevant objects in the world across saccades. This spatial constancy has been attributed to a process known as predictive remapping, in which immediately prior to a saccade, visual neurons respond to stimuli falling in their future, rather than current retinotopic receptive field. Although this mechanism has been extensively demonstrated using invasive neurophysiological recordings in visual and oculomotor areas of primates (Duhamel et al., 1992; Nakamura and Colby, 2002), how predictive remapping manifests in the human brain is less clear. In this study, we used MVPA of EEG data to provide spatiotemporally resolved, neural evidence of saccadic remapping in humans.

By examining decoding results under different train–test combinations and a range of stimulus-saccade latencies, we demonstrated that the neural representation of a visual stimulus briefly presented before an impending saccade indeed encodes the predicted future retinotopic position of that stimulus. This remapping was tightly locked to stimulus onset and was most present when the stimulus was shown in a critical period centered on 120 ms before saccade onset. This shows that immediately before a saccade, the visual system predictively represents visual objects in their anticipated future retinotopic positions, which likely plays a key role in maintaining visual stability across eye movements. In addition, through examination of central saccade trials in the Short Bin, we show that the pre-saccadic retinotopic stimulus location is not lost due to remapping. Above-chance decoding at overlapping timepoints in central saccade trials and peripheral saccade trials suggests simultaneous representation of both the pre-saccadic retinotopic location and the remapped location. In addition, pre-saccadic location information is still available after saccade offset. This supports the idea of a slow switch of attention from the original retinotopic position pre-saccade to the relevant spatiotopic position, post-saccade.

The importance of stimulus timing relative to saccade onset suggests that corollary discharge (CD) is the driving force behind this predictive process. We found above-chance decoding of the remapped location only when the stimulus was presented in a narrow window centered on 120 ms before saccade onset. This is when saccade preparation occurs and, by consequence, when the CD signal is available (Sommer and Wurtz, 2004, 2008; Crapse and Sommer, 2008; Wurtz, 2008; Cavanaugh et al., 2016). The significance of the short SSOA for remapping is also in line with numerous behavioral studies that found facilitation of behavior at the remapped position when the stimulus was presented immediately before a saccade (Melcher, 2007; Zirnsak et al., 2011; Panichi et al., 2012; Li et al., 2016; He et al., 2018). Our results provide a neural explanation for this behavioral enhancement by showing that the remapped stimulus location is predictively activated in anticipation of the post-saccadic stimulus.

Under hierarchical models of visual processing, after an initial feedforward sweep of visual information, higher level areas, such as the frontal eye fields, use corollary discharge to provide spatiotemporally precise predictions for lower levels within the visual hierarchy (Schall et al., 1995; Stanton et al., 1995; Grzeczkowski et al., 2020). This ensures that visual stimuli are aligned with their expected post-saccadic retinotopic location despite saccades interrupting the typical flow of visual information. We expect that when the peripheral stimulus entered the first stages of visual processing, information about its current (pre-saccadic) position was combined with information from the CD signal to predict the future (post-saccadic) scene. By activating neuronal receptive fields at the post-saccadic retinotopic location, information can be gathered about the post-saccadic scene to minimize interruptions caused by the saccade. As shown by the temporal overlap in above-chance decoding for central saccade trials (Fig. 6*b*) and peripheral saccade trials (Fig. 4*c*), such information gathering from the post-saccadic location does not disrupt the allocation of resources to the pre-saccadic stimulus position. This supports the dual-spotlight theory which proposes that attention is divided across two locations at saccade offset (Golomb, 2019). When the saccade occurred with a longer latency, we instead saw a pattern of decoding very similar to control trials, indicating that the stimulus was being processed at its true retinotopic position (on the opposite side of the screen) rather than the remapped one. The fact that we found no evidence of remapping with longer saccade latencies indicates that the brain must be in a state of motor preparation when the visual information enters the system to elicit such a predictive shift.

The temporal precision afforded by our analysis approach revealed not only the time window during which stimuli presented before a saccade are remapped but also when the remapped information about those stimuli is available in the EEG signal. Roughly speaking, remapping occurred for stimuli presented ∼120 ms before saccade onset, but peak decoding was achieved ∼184 ms after stimulus onset (i.e., once the eyes were already in flight). Using the median saccade offset as a marker (251 ms post-stimulus onset; peripheral saccade trials), this suggests that predictive remapping as a neural process starts before a saccade, but continues into the trans- and post-saccadic period. It is important to note that the neural response we observed cannot simply be attributed to normal visual processing, since the stimulus was removed from the screen before saccade onset (contrary to some previous studies where the stimulus remained on screen, e.g., Parks and Corballis, 2010; Edwards et al., 2018). This means that there was no direct stimulation of the classical neuronal RFs representing the locations on which the classifiers were trained (i.e., one of the four locations around central fixation). As outlined by Duhamel et al. (1992), the post-saccadic response is reflective of an expectation that the stimulus will fall in the neuronal RF after the eyes arrive at the saccade endpoint. These results are compatible with animal neurophysiological research which found that stimuli presented in close proximity to the saccade were remapped after the saccade (Kusunoki and Goldberg, 2003; Neupane et al., 2016).

The finding that the pre-saccadic retinotopic location and the remapped location are decodable at overlapping timepoints is in line with the dual-spotlight theory of attention. It is proposed that neurons with RFs encompassing the relevant post-saccadic position (remapped location) begin to fire in anticipation of the stimulus while simultaneously, neurons with RFs at the pre-saccadic location of the stimulus continue to fire, even after the saccade has been executed (Golomb et al., 2010; Golomb, 2019). This has been demonstrated behaviorally as attentional facilitation at two locations, simultaneously (Golomb et al., 2008, 2011, 2014). Interestingly, the timepoints at which both the remapped location and the pre-saccadic retinotopic location can be decoded are before saccade offset, likely when the eyes are in motion. While this indicates the simultaneous encoding of two retinotopic locations in the trans-saccadic period, it should be noted that the decoding evidence at each location comes from separate sets of saccade trials, i.e., peripheral saccade trials and central saccade trials. In other words, EEG data from peripheral saccade trials were used as a test set to check for above-chance decoding at the remapped location, while central saccade trial data were used to test decoding at the pre-saccadic retinotopic location. Thus, while above-chance classification at overlapping timepoints indicates attentional facilitation at both locations simultaneously, this was not directly measured. Ideally, the retinotopic pre-saccadic and post-saccadic stimulus positions from the same trial could be tested. This was not possible with the current design as classifiers were trained only on the four locations directly around current fixation and not on the four retinotopically peripheral locations. This means that, when testing peripheral saccade trials, the remapped location overlapped with trained retinotopic positions but the stimulus' pre-saccadic location did not, while the opposite was true for central saccade trials, in which the pre-saccadic location overlapped with trained positions but the remapped location did not. Future studies employing similar methods should take this into consideration. We did, however, find evidence for the retinotopic attentional trace, in the continued representation of the pre-saccadic retinotopic position after saccade offset in central saccade trials. This is in line with MEG results in which spatial frequency could be decoded from the pre-saccadic location up to 100 ms after saccade offset (Fabius et al., 2020).

**Figure 7.**
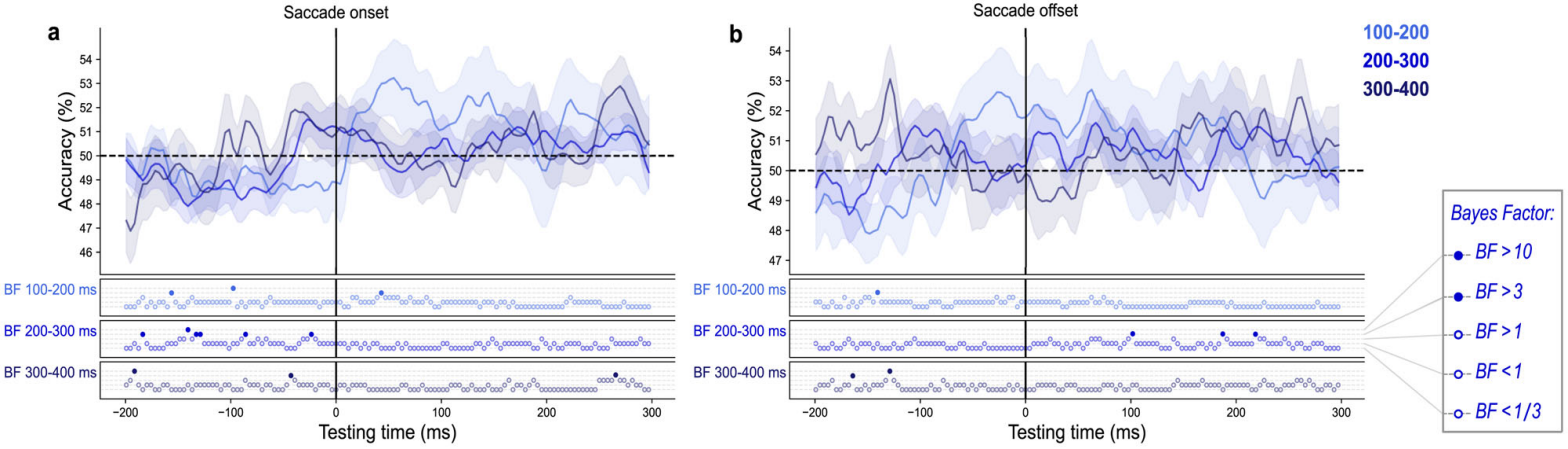
Saccade-locked analysis of peripheral saccade trials. Average performance of classifiers trained on the peak timepoint of fixation trials and tested on peripheral saccade trials locked to (***a***) saccade onset and (***b***) saccade offset. The saccade trials are split according to saccade latency relative to stimulus onset (i.e., 100–200 ms, 200–300 ms, 300–400 ms after stimulus onset). The BFs below the plot indicate the timepoints at which there was substantial evidence in favor of the alternative hypothesis, i.e., decoding above-chance (filled circles) or a lack of evidence (open circles).

Our results indicate that the timing of remapping is dictated by when sensory information enters the system. This is evidenced by above-chance decoding that is dependent on stimulus presentation timing. When we repeated the same analyses using EEG epochs time-locked to the saccade, rather than stimulus onset, there was very little evidence of remapping (Fig. 7). One explanation for this could be the use of ICA eye movement artifact removal. While this is a popular method used for the removal of corneoretinal artifacts (Dimigen et al., 2011; Katz et al., 2020; Huber-Huber and Melcher, 2023), it has been criticized by some who suggest that peri-saccadic effects may be lost in the process (Huber-Huber et al., 2016). However, another possible explanation is the temporal misalignment of remapping signals. Temporal alignment to the saccade (rather than the stimulus) causes remapping signals to misalign such that they are smeared in the average. The fact that remapping is locked to the stimulus and not the saccade seems, at first glance, to contradict the idea that remapping is a process driven by a motor action. However, the current analysis exploits the fact that the onset of a visual stimulus triggers waves of activity, both feedforward and feedback (Goddard et al., 2016; Dijkstra et al., 2020; Aggarwal et al., 2022), that change the activity pattern across the scalp as they propagate through the visual hierarchy (Carlson et al., 2011). We trained classifiers using the stimulus evoked activity at a particular timepoint during visual processing. When we time lock the test data to the saccade, visual information will have reached different processing stages in different trials leading to across-trial desynchronization. This means that the classifiers likely identified remapped stimulus information at different timepoints across trials, which is then lost in the grand average. By time-locking to the stimulus onset, we show that presenting the stimulus during a state of motor planning changes the course of the sensory processing such that it continues along the visual hierarchy as if it were presented at its remapped location.

This interpretation is consistent with findings reported by Edwards et al. (2018), who showed that, after saccade landing, it was possible to discriminate a face stimulus from a house stimulus briefly presented in the periphery before a saccade. Contrary to our result, they observed significant above-chance decoding when locking to saccade offset. However, in their study the time between stimulus onset and saccade onset was fixed so that the propagation of feedforward visual information had always reached the same point before the initiation of a saccade. In the present study, the time between stimulus onset and saccade onset was deliberately varied, such that there was a greater variability of SSOAs within each saccade bin. If SSOA is variable, and if predictive shifts are driven by the CD signal, then the level at which the CD signal interacts with the visual signal may differ from trial to trial, masking any saccade-locked effects. Another important difference between the current study and that by Edwards et al. (2018) is the stimulus type. Purely spatial information, as examined in the current study, and category information, as in Edwards et al. (2018), are processed in a neurally distinct manner. Stimulus category falls under high-level vision and is assumed to be processed in an invariant way, such that neurons will continue to fire in response to a specific category despite changes in spatial position, size, or color (Quiroga et al., 2005; DiCarlo and Cox, 2007). By locking to the stimulus, we examine how presenting the stimulus in a state of motor preparation transforms its representation in the visual cortex.

Previous studies have argued in favor of attentional shifts as an explanation for saccadic remapping (Melcher and Colby, 2008; Cavanagh et al., 2010; Jonikaitis et al., 2013; Wolf and Schütz, 2015; Rolfs and Szinte, 2016). In the current experiment, both exogenous (abrupt stimulus onset) and endogenous attention (task-relevant stimulus) were cued, meaning attention was engaged on all trials. However, contrary to previous behavioral studies supporting attentional remapping, we did not find remapping when the stimulus was presented significantly before saccade onset. It has been found that presenting an attentional cue considerably before saccade onset (which would correspond to the Middle and Long Bins in the current study) leads to pre-saccadic sensitivity at the remapped location, while presenting the cue close to saccade onset leads to no remapping (Szinte et al., 2018). This suggests possible mechanistic differences between predictive remapping of attention (which has primarily been measured in humans using psychophysical approaches) and spatial remapping of neuronal receptive fields (which has primarily been measured in animal models). Task design may also play a role in this discrepancy. The current study employed a design in which fixation trials, saccade trials, and control trials were randomly interleaved, meaning participants could not anticipate when a saccade should be initiated. In contrast, other studies included only saccade trials, meaning that stimulus presentation was always accompanied by a saccade (Szinte et al., 2018). Perhaps, participants used this predictability to prepare a saccade in advance of the saccade cue. This may explain why such studies find remapping despite the attentional cue being presented outside of the normal saccade preparation range (∼200 ms before saccade onset; Fischer and Ramsperger, 1984). In the current study, attention was always allocated to the stimuli, such that we cannot dissociate between attentional remapping and spatial RF remapping. Further research dissociating attention, for example, by including trials in which attention is directed toward a location away from stimulus presentation location (similar to that implemented while recording from monkey V4; Marino and Mazer, 2018), will therefore be necessary to characterize the role of attention in saccadic remapping.

In conclusion, we provide strong neural evidence in humans of predictive remapping of visual spatial information across saccades. Moreover, we found that the timing of remapping was tightly locked to stimulus presentation, occurred only for stimuli presented in a narrow window immediately preceding a saccade, and continued while the eyes were in motion. In addition, we found evidence for retinotopically lingering attention, whereby the pre-saccadic retinotopic location was decodable after saccade offset. Thus, predictive remapping may ensure visual constancy across eye movements by enabling the visual system to represent the pre-saccadic stimulus at its post-saccadic retinotopic position while ensuring information from the pre-saccadic location remains available for a short time.

## Data Availability

All experiment scripts, data, and analysis code will be made available on the Open Science Framework (https://osf.io/uqpxy/).
